# Detection and Identification of *Bacillus anthracis*: From Conventional to Molecular Microbiology Methods

**DOI:** 10.3390/microorganisms8010125

**Published:** 2020-01-16

**Authors:** Aleksandra A. Zasada

**Affiliations:** Department of Sera and Vaccines Evaluation, National Institute of Public Health—National Institute of Hygiene, Chocimska 24, 00-791 Warsaw, Poland; azasada@pzh.gov.pl

**Keywords:** *Bacillus anthracis*, detection, identification, rapid tests, microbiological methods, molecular microbiology methods, biosensors, molecular markers

## Abstract

Rapid and reliable identification of *Bacillus anthracis* is of great importance, especially in the event of suspected deliberate release of anthrax spores. However, the identification of *B. anthracis* is challenging due to its high similarity to closely related species. Since Amerithrax in 2001, a lot of effort has been made to develop rapid methods for detection and identification of this microorganism with special focus on easy-to-perform rapid tests for first-line responders. This article presents an overview of the evolution of *B. anthracis* identification methods from the time of the first description of the microorganism until the present day.

## 1. Introduction

*Bacillus anthracis* is an etiological agent of anthrax—a serious infectious disease in animals and humans with a very long history. It is believed that the first record of anthrax is in the Bible in Exodus, chapters 7–9. The disease was well known by the Greeks and Romans as its typical symptoms were described in the writings of Homer (ca. 1000 B.C.), Hippocrates (ca. 400 B.C.), Virgil (70–19 B.C.), and Galen (ca. 200 A.D.). Anthrax was a major worldwide cause of death for animals until the end of the 19th century [1,2]. Also, human cases occurred frequently in persons that had come into contact with sick and dead animals and with animal-derived products contaminated with *B. anthracis* spores. For example, in the 17th century, a widespread anthrax epidemic in Europe, called “black bane”, was related to a large number of deaths among animals and humans. It was estimated that 60,000 people died due to the *B. anthracis* infection in 1613 alone [1,3].

Due to the high economic impact of anthrax epidemics in livestock as well as the seriousness of human infections, the disease attracted the attention of microbiologists. Also, it is probably for these reasons that *B. anthracis* became the basis for the development of bacteriology and microbiological diagnostics. The 19th century was especially fruitful in terms of the study of anthrax. In 1823 Barthelemy demonstrated the infectiousness of the disease; in 1838 Delafond observed the bacilli bacteria for the first time; in 1863-1864 Davaine demonstrated the transmissibility of anthrax; and in 1864 Tiegel and Klebs demonstrated that the infectivity of infectious material was lost on filtration through cay filters. Robert Koch also studied anthrax bacilli and formulated his famous Postulates in 1877 proving that *B. anthracis* was the cause of anthrax [2]. Moreover, Robert Koch’s observation that *B. anthracis* produced spores under starvation conditions together with the observation that the spores were extremely resistant to a variety of physical and chemical treatments helped in the understanding of the epidemiology of the disease and the formulation of efficient rules for the prevention of dissemination of the disease. It also highlighted the possibility that *B. anthracis* could become a biological weapon in the following decades [1].

The infective form of *B. anthracis* is spores. The spores germinate in a host organism (human or animal) to produce the vegetative forms which rapidly multiply and express the anthrax toxins and the poly-D-glutamic acid capsule—the major pathogenicity factors coded by genes located on the virulence plasmids pXO1 and pXO2, respectively. The anthrax toxins consist of three synergistically acting proteins: protective antigen (PA), edema factor (EF), and lethal factor (LF). PA in combination with EF forms the edema toxin and PA in combination with LF forms the lethal toxin. The toxins are responsible for the characteristic signs and clinical symptoms of the disease whereas the poly-D-glutamic acid capsule protects the bacterium from phagocytosis [4].

In the 20th century anthrax was still one of the most significant diseases globally and the annual incidence of human cases of anthrax worldwide, estimated by the World Health Organization (WHO) in 1958, was 20,000–100,000 [1]. However, due to the development of an anthrax vaccine for animals and improvement of hygienic conditions for farmers and workers using animal-derivates, anthrax became sporadic in developed countries in the second half of the 20th century. Interest in anthrax, with special focus on detection and identification of *B. anthracis* in environmental and clinical samples, increased again in 2001 after the bioterrorist attacks in the USA called Amerithrax [5]. It was also at this time that rapid and easy-to-perform tests for use by first-line responders (e.g., firefighters, soldiers, police officers, and emergency medical personnel), were most needed. Together with the development of sophisticated microbiological and molecular biology methods, this situation resulted in a rapid increase in scientific publications concerning new methods for *B. anthracis* detection and identification; however, many were verified as being related to unspecific reactions.

## 2. Challenges for *B. anthracis* Identification

The difficulties in identification of *B. anthracis* are related to the high phenotypic and genetic similarity of this species to *Bacillus cereus* and other closely related species. The similarity is so high that some researchers have considered *B. anthracis* to be a pathogenic variant of *B. cereus*. This thesis was probably presented for the first time by Smith and co-workers in 1946 [6] and the scientific discussion on this subject has been ongoing for several decades. Other genetically, closely related species of *Bacillus* genus that are widely distributed in the environment include *Bacillus thuringiensis*, *Bacillus mycoides, Bacillus pseudomycoides,* and *Bacillus weihenstephanensis.* The genome similarity between *B. anthracis* and *B. cereus, B. thuringiensis, B. mycoides, B. pseudomycoides*, and *B. weihenstephanensis* is so significant that all these species have been included in one bacterial group called *B. cereus* Group *sensu lato* [7,8,9]. Even *B. anthracis* virulence plasmids or their parts may be transferred to closely related species [10,11,12,13].

Challenging are also differences between clinical and environmental samples containing *B. anthracis* and the approach taken should depend on the type of samples being examined. Whereas vegetative cells are expected in fresh clinical samples, in environmental samples spores are expected, which are the infective form of the bacteria. On the one hand antigen content of vegetative cells and spores differs, which must be considered when antigen-based approaches are used [14]. On the other hand, spores are highly resistant to adverse environmental conditions including temperature, radiation, common disinfectants, and many other chemicals and viable spores might still be present in extracted DNA samples causing the risk of laboratory-acquired infection [15]. Even the filtration of DNA samples might not provide 100% removal of viable spores from the samples [16].

Moreover, environmental samples may contain various microorganisms and their spores, as well as various unidentified substances that can interfere with components of a test causing the reduction of the sensitivity and even inhibition of the reaction or false-positive results due to cross-reactivity.

## 3. Conventional Microbiological Methods

Application of conventional microbiological methods for bacterial identification is always related to the necessity of culture of the microorganisms. Conventional methods of *B. anthracis* identification include growth on selective media, lack of hemolysis, lack of motility, capsule staining, gamma phage lysis, ‘String-of-pearls’ reaction, and susceptibility to penicillin.

Conventional microbiological methods are the gold standard for identification of bacteria but might be misleading in the case of *B. anthracis*. This is because many *B. anthracis* phenotypic features, such as lack of motility, lack of hemolysis, gamma phage lysis, and susceptibility to penicillin, can be exhibited by *B. cereus* isolates [17,18,19,20,21]. On the other hand, hemolytic *B. anthracis* strains, as well as ones resistant to penicillin and gamma phage, have also been isolated [17,18,22,23,24]. Moreover, application of conventional microbiological methods requires culture and handling of living microorganisms, which is always related to the risk of laboratory-acquired infections.

### 3.1. Selective Media

Polymyxin-Lysozyme-EDTA-Thallous acetate (PLET) agar is the most common selective medium for *B. anthracis*. It is heart infusion agar supplemented with polymyxin B, lysozyme, ethylene diamine tetra acetic acid (EDTA), and thallous acetate. PLET agar seems to be the most effective medium for isolation of *B. anthracis* from mixtures containing other spore-forming bacilli. Colonies of *B. anthracis* on PLET agar appear small, white, domed, and circular [25]. However, thallous acetate is highly toxic and therefore the use of PLET medium is excluded in some countries due to work and safety regulations [26].

Also, semi-selective media are available. R & F Anthrax Chromogenic Agar (ChrA) is a semi-selective medium which contains cycloheximide, polymyxin B, and X-Indoxyl-choline phosphate (X-CP). X-CP detects the enzyme phosphatidylcholine phospholipase C (PCPLC) which is produced by *B. anthracis* but also *B. cereus* and *B. thuringiensis*. *B. anthracis* colonies on ChrA appear rough, with a ground-glass texture, and are cream to pale teal-blue after 24 h of incubation while turning teal-blue with a large white rim after 48 h. *B. cereus* and *B. thuringiensis* colonies are dark teal-blue within 24 h. This is because of differences between these *Bacillus* species in the rate of PCPLC production. The rate of production of this enzyme is about 10 times lower for *B. anthracis* than for *B. cereus* and *B. thuringiensis* [20,27]. Anthrax Blood Agar (ABA) contains cycloheximide, polymyxin B, trimethoprim, and sulfamethoxazole as a selective supplement. *B. anthracis* colonies on ABA are non-hemolytic, white, creamy or gray-colored. Cereus Ident Agar and Chromogenic Bacillus Cereus Agar contain polymyxin B, trimethoprim, and the chromogenic substrate 5-bromo-4-chloro-3-indolyl-ß-glucopyranoside. The chromogenic substrate is cleaved by the enzyme ß-glucosidase produced by the majority of *Bacillus* spp. and results in the formation of blue/green colonies. However, *B. anthracis* colonies on these media are white to creamy [26].

### 3.2. Capsule Staining

The presence of capsulated cells of *B. anthracis* might be confirmed by M’Fadyean staining with polychrome methylene blue or India ink staining. Polychrome methylene blue is a mixture of methylene blue and other homologs, such as azure A and azure B, which are produced during methylene blue storage by its oxidation. After staining, the *B. anthracis* cells are visible under a microscope as blue-black, square-ended bacilli surrounded by pink capsules. However, the *B. anthracis* capsule loses its affinity for methylene blue during putrefaction and therefore the capsule might not be visible in M’Fadyean staining when samples from partially decomposed carcasses are investigated [4].

The capsule can also be exposed using India ink. The capsule is visible as a transparent halo around the bacterial cells. However, the method seems to be less sensitive than M’Fadyean staining [4].

### 3.3. The Phage Lysis Test

In 1931 Cowles described a bacteriophage which was active on *B. anthracis* but not specific for that species [28]. Twenty years later, McCloy isolated a bacteriophage which was active for 171 *B. anthracis* strains and 2 of 54 *B. cereus* strains tested. However, the phage failed to produce lysis of smooth variants of *B. anthracis* [29]. Finally, Brown and Cherry isolated gamma (γ) phage which had the ability to lyse *B. anthracis* (including smooth forms) but not *B. cereus* [30]. The γ phage is still used for *B. anthracis* identification [4,31]. The specificity of this test is estimated at 96%, as a small number of other *Bacillus* spp. and non-*Bacillus* spp. environmental strains revealed to be susceptible to γ phage [32].

### 3.4. ‘String-of-Pearls’ Reaction

The ‘string-of-pearls’ reaction was described by Jensen and Kleemeyer in 1953 [33]. It is based on the phenomenon that *B. anthracis* growing on a solid medium, such as Tryptose Agar, containing 0.05–0.5 units of penicillin per ml at 37 °C for 3–6 h, forms large, spherical cells occurring in chains which resemble a string of pearls. The test was used historically and currently is not recommended.

## 4. DNA Amplification-Based Methods

The invention of the DNA amplification method using the polymerase chain reaction (PCR) by Kary B. Mullis in 1983 provided new possibilities for the identification of microorganisms. This was also reflected in the identification of *B. anthracis*.

DNA amplification-based methods have some advantages, such as a lack of the necessity to culture the microorganisms and the possibility to test inactivated samples, which make these methods safer than conventional methods.

### 4.1. PCR and Real-Time PCR

The principle for DNA amplification-based identification of microorganisms is the selection of appropriate genetic markers. The most widely used genetic markers for *B. anthracis* identification are located on anthrax virulence plasmids pXO1 and pXO2. There are usually genes coding components of anthrax toxin (protective antigen, edema factor, lethal factor) located on pXO1 and genes coding the capsule located on pXO2 (e.g., [4,34,35]).

The anthrax toxin and the capsule are virulence factors and their detection also provides information about the pathogenic properties of the strain. In general, the lack of any of the virulence plasmids decreases the pathogenic properties of the *B. anthracis* strain [2,36,37]. However, because the plasmids can be lost by *B. anthracis* as well as transferred to other bacilli from the *B. cereus* Group *sensu lato*, researchers have tried to identify chromosomal markers restricted to *B. anthracis* [10,19,36,38,39]. Selection of a chromosomal marker appropriate for *B. anthracis* identification has proved to be challenging. A lot of proposed markers have transpired to be unspecific. As an example, the following chromosomal markers can be mentioned: Ba813 [40,41], BA-5449 [42,43], ORF *vrrA* [44,45], and *rpoB* [46,47]. Application of DNA sequencing enables detection-specific single nucleotide polymorphisms (SNPs) specific for *B. anthracis* in selected chromosomal markers. However, the use of DNA sequencing is complicated in the field. Therefore, the use of various molecular probes, HRM as well as PCR-RFLP, for detection of specific point mutations has been proposed; for example, PCR-RFLP of SG-850 marker (also called SG-749 due to its amplicon size in *B. anthracis*) [48] and RSI-PCR of *plcR* marker [49]. According to current data, both methods are considered to be specific, but require time-consuming post-PCR manipulations. Easterday et al. [50] proposed the application of minor groove binding (MGB) probes specific for the point mutation in the *plcR* gene of *B. anthracis* and real-time PCR. However, a delayed positive signal can be obtained for some *B. cereus* strains, which limits the usefulness of the assay.

Nevertheless, the detection of SNPs in the field by first-line responders in cases of suspected anthrax release is challenging. Currently, only two types of tests are available on the market for field application by first-line responders: rapid lateral flow assays described further in the article and PCR systems for field screening. PCR systems for field screening of *B. anthracis* include: JBAIDS (BioFire Defense Inc., Salt Lake City, UT, USA), FilmArray (BioFire, Salt Lake City, UT, USA), RAZOR EX (BioFire, Salt Lake City, UT, USA), T-COR 4 and newer version T-COR 8 (Tecracore, Rockville, MD, USA), POCKIT (GeneReach USA, Lexington, MA, USA), and Bio-Seeq PLUS (Smiths Detection, Edgewood, MD, USA). All the systems are based on real-time PCR technology and can be used with dedicated reagent kits. FilmArray enables investigation of one sample at a time for 27 genetic targets of 17 pathogens (BioTreat Panel), including three targets for *B. anthracis*: pXO1, pXO2, and chromosomal. RAZOR EX enables analysis of one sample at a time for simultaneous testing of ten pathogens (Ten Target Screen Kit), including a pXO2 assay for *B. anthracis*. T-COR 4 and T-COR 8 enable analysis of up to four and eight samples at a time, respectively. For *B. anthracis* detection, pXO1 and pXO2 assays are available (two separate kits for T-COR 4 and one combined kit for T-COR 8). Reagent kits for the systems mentioned above are available in a freeze-dried format and can be stored at room temperature. POCKIT enables analysis of up to eight samples at a time and for *B. anthracis* detection three assays targeting pXO1, pXO2, and chromosomal PL3 markers are available. The reagents are produced in a dry format, but recommended storage temperature is 4 °C. The reaction time is about one hour for all the systems [51,52,53,54].

Real-time PCR has some advantages compared to PCR, such as the possibility of quantification of the synthesized product and the lack of post-PCR manipulations for detection of results. However, both techniques require precise temperature changes and therefore dedicated apparatuses are necessary. This disadvantage might be overcome by isothermal DNA amplification methods.

### 4.2. Isothermal DNA Amplification Methods

DNA amplification in isothermal conditions is a very promising tool and several methods have been described, such as recombinase polymerase amplification (RPA), helicase-dependent amplification (HDA), loop-mediated isothermal amplification (LAMP), rolling circle amplification (RCA), isothermal and chimeric primer-initiated amplification of nucleic acids (ICAN), strand displacement amplification (SDA), single primer isothermal amplification (SPIA), and polymerase Spiral Reaction (PSR).

Application of LAMP for *B. anthracis* identification was described by Qiao et al. [55] using *pag* (pXO1), *capB* (pXO2), and Ba813 (chromosomal) markers, and by Kurosaki et al. [56] using *pagA* (pXO1), *capB* (pXO2), and *sap* (chromosomal) markers. In the LAMP assay, a set of six primers for each marker is needed and the amplification is conducted by a strand-displacing DNA polymerase for about an hour at 60–65 °C. A result of the reaction is a mixture of various sized amplicons, visible on the agarose gel as a ladder-like pattern of bands, because the LAMP products consist of several inverted-repeat structures. The primers designed by Kurosaki et al. have subsequently been applied by other researchers who have revealed very limited specificity of the assay [57].

The RPA assay for *B. anthracis* detection was proposed by Euler et al. [58]. In the assay, a target sequence should be 80–400 bp in size. A pair of primers is used for the reaction and the amplification occurs thanks to the action of three proteins: a strand-displacing polymerase, a recombinase, and a single-stranded DNA binding protein. The reaction is conducted for about 30 min at 38 °C. Euler et al. [58] designed RPA primers for *pagA* (pXO1) and *capC* (pXO2), whereas Bentahir et al. [59] designed RPA primers for BA_5345 (the chromosomal marker), *lef* (pXO1), and *capA* (pXO2). High specificity of the results was shown. On the other hand, Zasada et al. [57] (for RPA assays) applied primers for *pagA* and *capB* commonly used in PCR and revealed high specificity for *pagA* markers and limited specificity for *capB* markers.

In the HDA, a strand-displacing polymerase, a single-stranded DNA binding protein, a helicase, and a pair of primers are involved in the reaction, which is conducted for about 90 min at 65 °C. Zasada et al. [57] tried to apply the assay for *B. anthracis* identification based on *pagA* and *capB* markers but, due to the small size of the amplification products, ranging from 80 to 120 bp, a high level of cross reactivity with other members of the *B. cereus* Group was observed.

The amplicons obtained in the above-mentioned isothermal amplification methods can be visualized by conventional agarose gel electrophoresis as well as with the use of lateral flow dipsticks [57]. Also, application of colorimetric methods for LAMP amplicon visualization was described with the use of fluorescent DNA intercalating dyes (e.g., SYBR Gold, EvaGreen, and QuantiFluor), metal ion indicators (indication of Mg^2+^), such as hydroxynaphthol blue (HNB) and calcein, and pH-sensitive dyes, such as neutral red and phenol red [55,60,61,62]. Some of these are added to the pre-reaction solution; and therefore, the reaction progress can be observed directly in real time.

## 5. Antigen-Based Identification Methods

An alternative approach for detection of *B. anthracis* is antigen detection by immunoassay. Antibodies for various antigens have been selected, such as glycoprotein BclA, which is a major component of the exosporium of *B. anthracis* spores [63], as well as oligosaccharide epitopes of BclA [63,64], extracellular antigen 1 EA1, which is a major S-layer component of *B. anthracis* [14,65], the protective antigen PA, which is a component of anthrax toxin [66] and poly-D-glutamic capsule [67]. The selection of an appropriate target antigen for the assay depends on the type of tested samples because different antigens are exposed on the surface of *B. anthracis* vegetative cells and spores. Also, cross reactivity with other members of *B. cereus* Group has been observed [63,64,68].

Immunoassays applied for *B. anthracis* identification include flow cytometry assays combined with fluorescein-labeled antibodies [69] and FRET (Förster resonance energy transfer) [70,71], ELISA [63], Luminex assay [64], a magnetic particle fluorogenic immunoassay (MPFIA) [72], ABICAP immunofiltration, a lateral flow assay [65,73,74], biosensors [75], and many others.

Lateral flow assays (LFA) are the second group of tests commercially available for first-line responders. These tests are easy to perform and no equipment is required. In the assay, the capture-labeled antibody binds to its target antigen and flows along a chromatography strip by capillary force. Then, the antibody–antigen complexes and free-labeled antibodies are captured by secondary antibodies immobilized at two regions on the strip forming a test line and a control line, respectively [76]. Results of the test can be read by eye within 15 min. Additionally, some manufacturers offer portable reading devices [77]. However, comparison of lateral flow tests reveals very low sensitivity, in practice lower than the sensitivity declared by the manufacturer, and in the case of complex samples, such as environmental and clinical samples, the lateral flow assay often suffers from interference from particle components in the tested samples [73,76].

A luminescent adenylate cyclase assay for detection of active edema factor in *B. anthracis* culture was developed [78]. The assay is based on the EF-dose response decrease in luminescence, which may be specifically reverted by anti-EF antibodies. The assay enables not only identification of *B. anthracis* in cultures but also gives information about expression of one of the key pathogenicity factors of these bacteria.

Immunological methods are also used for anthrax diagnostics to identify cellular immunity developed post infection. However, these methods are useful for retrospective diagnostics because several days are needed to develop specific antibodies during the infection. The most common method for detection and quantification of an antibody immune response is enzyme-linked immunosorbent assay (ELISA). The assay was developed for the detection of antibodies against various antigens, such as protective antigen [79], capsular antigen [80], and lethal factor [81]. Chitlaru et al. [82] generated a list of 84 in vivo-expressed *B. anthracis* immunogens that can be used in serodiagnostics and vaccine development.

Recently, Bar-Haim et al. [83] proposed a novel approach of diagnostics based on cellular reactivity of host cells with specific pathogen antigens. In this cell-based activation immunoassay the presence of interferon-gamma-producing (IFNγ-producing) activated cells might be detected in peripheral blood samples 3–5 days earlier than serum antibodies.

As an alternative to detection of specific antibodies, detection of *B. anthracis* biomarkers in clinical samples has been proposed [84]. The biomarkers have been identified by the proteomic assay and might be detected in serum samples at 3 to 6 h post-infection in an animal model. However, detection of the biomarkers could be achieved only after removal of highly abundant serum proteins by chromatography.

## 6. Biosensors

Currently, biosensors are the most promising technology for rapid, highly sensitive and specific detection and identification of *B. anthracis* with the potential for their use as portable devices. A biosensor comprises biological recognition molecules immobilized on a signal transducer which transforms the signal into readable output. According to the signal transduction, biosensors can be classified as electrochemical (amperometric, potentiometric, and conductometric), optical, piezoelectric, and thermal sensors [85]. The majority of developed biosensors for *B. anthracis* identification rely on antibodies (immunosensors) or nucleic acid probes (genosensors) as the recognition molecules. The time necessary for obtaining results varies from a few minutes to a few hours.

### 6.1. Nucleic Acid Probes and Genosensors

Nucleic acid probes bind with specific DNA markers. DNA markers used for *B. anthracis* detection with biosensors are similar to those described above for DNA amplification-based methods.

For example, Raveendran et al. [86] developed a DNA biosensor based on the cyclic voltammetry (CV) redox peaks. In the biosensor, a self-assembled layer of tiol-linked *pagA* specific probe and mercaptohexanol (MCH) immobilized on the gold electrode. Hybridization of target DNA to the probe results in CV peak current and potential change. Ziółkowski et al. [87] also developed a voltametric biosensor but it contains a stem-loop probe linked to the gold electrode for recognition of the *pagA* marker. The presence of the *pagA* marker in the sample causes opening of the loop of the probe by hybridization with the target marker and a change in registered electrochemical signal. Hao et al. [88] applied a quartz crystal microbalance (QCM) biosensor for detection of BA813 chromosomal markers and *pagA* markers. In this biosensor, a thiol-modified DNA probe is attached to the QCM gold surface. Hybridization of the probe with the target marker amplified in asymmetric PCR causes a mass increase and a decrease in the resonance frequency. An example of optical DNA biosensors is a biosensor based on a photonic crystal structure used in a total-internal-reflection (PC-TIR) sensor and applied for detection of *lef* (pXO1) marker of *B. anthracis*. In this assay, the biotinylated probe is immobilized on the sensor via biotin–streptavidin interactions. The addition of target DNA causes hybridization with the probe and a significant increase in the resonance wavelength [89].

### 6.2. Antibody Probes and Immunosensors

Antibody probes bind with *B. anthracis* antigens. Antigens used for *B. anthracis* detection with biosensors are similar to those described above for immunoassays.

An example of this type of biosensor is a metal-enhanced electrochemical immunosensor which comprises specific antibodies self-assembled onto a gold quartz crystal electrode via cystamine bond. A solid-phase monolayer of silver underpotentially deposited onto the cystamine modified-Au-electrode surface is used as the redox probe. When the antibody-modified electrode is exposed to spores, complexes of the antigen–antibody are formed on the surface of the electrode and variation in the redox current is observed [90]. Another approach involves the use of an ultrasensitive portable capillary biosensor (UPAC). The capillary is an enclosed system that acts as the flow cell, the waveguide, and the solid support for immobilized antibody probes. An evanescent excitation generates a signal from an antigen–antibody–fluorophore complex, which propagates along the capillary and is guided to the detector [90]. McGovern et al. [91] proposed application of lead magnesium niobate-lead titanate/tin (PMN-PT/Sn) piezoelectric microcantilever sensors (PEMS) for detection of *B. anthracis* spores. In this approach, the specific antibodies are immobilized on the platinum electrode of the PMN-PT layer. An amperometric immunoassay for detection of *B. anthracis* spores was developed by Waller at al. [92]. The assay includes immunomagnetic separation to capture the spores from a sample and amperometric measurement.

A quartz crystal microbalance sensor has also been used for antigen-based detection, similar to the DNA biosensor described above. In the assay designed by Hao et al. [93], antibodies were immobilized onto the gold electrode with protein A on a mixed self-assembled monolayer of 11-mercaptoundecanoic acid (11-MUA) and 6-mercaptohexan-1-ol (6-MHO) as an adhesive layer.

### 6.3. Aptamers and Peptide-Nucleic Acid Chimera Probes

It is worth mentioning an additional two types of biosensors: biosensors using aptamers as probes (aptasensors) and biosensors using peptide-nucleic acid chimeras (PNAs) as probes. Aptamers are single-stranded DNA or RNA oligonucleotides which are able to bind specifically to various targets, such as toxins and microorganisms, due to their unique three-dimensional structures. PNAs are synthetic particles that can be used for detection of DNA markers [75]. However, both approaches have not been widely used for *B. anthracis* identification yet, and very few papers have been published on this subject. Mazzaracchio et al. [94] developed an impedimetric aptasensor for single-step detection of *B. anthracis* spore simulants (*B. cereus* spores). Bruno et al. [95] developed an aptamer-magnetic bead-electrochemiluminescence assay for *B. anthracis* detection. Zhang et al. [96] proposed a sandwich-hybridization DNA detection system with PNA probes for detection of *pagA* markers.

Most of the biosensors developed and described in the scientific literature (both immunosensors and genosensors) have been tested on a very limited number of strains or even only on *B. anthracis* surrogates. Therefore, despite the initial high specificity of the assays, they might suffer from nonspecific reactions similar to those occurring in PCR and other antigen-based identification methods.

Table 1 presents the comparison of detection limit for selected *B. anthracis* detection assays.

## 7. MALDI-TOF MS

Matrix-assisted laser desorption ionization time-of-flight (MALDI-TOF) mass spectrometry (MS) has been applied for identification of microorganisms and, currently, is broadly used in medical and veterinary diagnostic laboratories (e.g., MALDI-TOF mass spectrometers with a database of mass spectra for most common microorganisms are available commercially). This method was also applied for *B. anthracis* identification from pure culture as well as directly from clinical and environmental samples [97,98,99,100]. However, despite the fact that the results of identification might be obtained within several minutes, false-positive and false-negative results are an important risk. Moreover, the method can only be used in the laboratory due to the significant size of the apparatus.

## 8. Conclusions

From the time of recognition of microorganisms as causative agents of diseases in humans and animals, *B. anthracis* has been a pathogen of special interest. A lot of effort has been made to find reliable methods for the identification of this species. Since the advent of the threat of bioterrorism, these efforts have focused on the development of rapid, sensitive, and specific assays. In particular, portable, easy-to-use devices for first-line responders are needed. Significant progress has been made in the rapidity of tests, where results might be obtained within a few minutes. Even using conventional PCR, results can be obtained within less than an hour [101]. However, despite progress in molecular biology and the rapid development of increasingly sophisticated methods, researchers still face the same problems (i.e., the similarity of *B. anthracis* to closely related species which are common in the environment). For these reasons, it is recommended to confirm positive results with additional tests. Also, limitations of the applied tests must be kept in mind, especially in terms of limit detection, to avoid mistakes caused by false-negative results for which public health, economic, and social consequences might be very high. False-negative results may affect the successful treatment of affected individuals. The treatment must be implemented at the early stage of infection to prevent death, especially in case of inhalational anthrax. On the other hand, the false-positive results may cause public fear in scenarios of bio-terror and tremendous calamity-response logistic and public implications.

## Figures and Tables

**Table 1:** Comparison of detection limit for selected *B. anthracis* detection assays.

Assay	Detected Marker	Limit of Detection	Reference
PCR	*cya*	10^3^ copies of plasmid/reaction 2 × 10^4^ spores/reaction	[34]
real-time PCR (FRET)	*rpoB*	1 pg DNA/reaction	[46]
real-time PCR (MGB)	*plcR*	100 fg DNA/reaction	[50]
RAZOR EX	*capB*	100 fg DNA/reaction	[51]
RAZOR EX	*pagA*	10 fg DNA/reaction	[51]
FilmArray	pOX1, pOX2	200 spores/reaction 2000 spores/mL	[52]
T-COR 4	pXO2	200 spores/reaction 2000 spores/mL	[52]
POCKIT	pXO2	200 spores/reaction 2000 spores/mL	[52]
LAMP	Ba813, *pag, capB*	10 spores/reaction 100 spores/2 mg powder	[55]
LAMP	*pag, cap, sap*	>10 fg/reaction3–6 CFU/reaction	[56]
RPA	*pagA*	100–1000 genome copies/reaction	[57]
LFA—Anthrax BioTreat Alert	-	10^7^ spores/mL	[73]
LFA—SMART II Anthrax Spores Detection Kit	-	10^8^ spores/mL	[73]
Piezoelectric biosensor	-	10^3^ CFU/mL	[93]
Voltametric biosensor	*pagA*	5.7 nM/reaction	[87]
Impedimetric aptasensor	-	3 × 10^3^ CFU/mL	[94]

Abbreviations: PCR, polymerase chain reaction; FRET, Förster resonance energy transfer; MGB, minor groove binding; LAMP, loop-mediated isothermal amplification; RPA, recombinase polymerase amplification; LFA, lateral flow assay; CFU, colony forming unit.
